# Hemorheological Alterations as a Driver of Microangiopathy in Diabetic Kidney Disease—The Role of Erythrocyte

**DOI:** 10.3390/ijms27083592

**Published:** 2026-04-17

**Authors:** Michael Garoufis, Christina Kostara, Sissy Foteini Sakkou, Sempastian Filippas-Ntekouan, Eleni Bairaktari, Vasileios Tsimihodimos

**Affiliations:** 1 Laboratory of Clinical Chemistry, Faculty of Medicine, School of Health Sciences, University of Ioannina, 45500 Ioannina, Greece; michalisgaroufis@gmail.com (M.G.); chkostara@gmail.com (C.K.); ebairakt@uoi.gr (E.B.); 2 Department of Internal Medicine, Faculty of Medicine, School of Health Sciences, University of Ioannina, 45500 Ioannina, Greece; sissy_sakkou@hotmail.com (S.F.S.); sebastienfilippas@hotmail.com (S.F.-N.)

**Keywords:** diabetic kidney disease, hemorheology, erythrocyte, microvascular dysfunction

## Abstract

Diabetic kidney disease (DKD) is a severe microvascular complication traditionally attributed to general metabolic derangement and genetic susceptibility. However, this classic pathophysiological approach overlooks the role of red blood cells in the development and the progression of the disease. Prolonged exposure to high blood glucose and oxidative stress compromises the cell’s membrane architecture and ionic homeostasis, resulting in altered rheological properties. By synthesizing these molecular-to-rheological pathways, this review establishes a novel pathophysiological framework for understanding DKD, repositioning erythrocytes to a primary catalyst of renal injury and a highly sensitive target for early diagnostic intervention.

## 1. Introduction

Diabetes mellitus (DM) represents a global pandemic affecting 580 million people aged 20–79 years old, with the number expected to rise by 45% over the next 25 years [1]. An alarming rise in the incidence of its complications has been observed since the early 2010s, with kidney failure taking the lion’s share [2]. Chronic kidney disease (CKD) attributed to DM is defined as the presence of albuminuria and/or persistent estimated glomerular filtration rate (eGFR) <60 mL/min/1.73 m^2^ or other manifestation of kidney damage, in the absence of any other evident cause [3].

Diabetic kidney disease (DKD) occurs in 20–40% of patients suffering from DM and represents a typical disease caused by the interaction between genes and environmental factors [4,5]. Metabolic alterations such as hyperglycemia, dyslipidemia and obesity accelerate kidney function decline in genetically susceptible individuals with key pathophysiological drivers including glomerular hyperfiltration, podocyte injury, tubulointerstitial inflammation and fibrosis [5]. However, beyond these well-established mechanisms, understanding the complete pathophysiology of diabetic kidney disease requires a critical pivot toward erythrocytes, redefining them from simple oxygen carriers into central mediators of microvascular damage and endothelial dysfunction [6,7,8]. Unlike conventional frameworks that view erythrocytes merely as secondary targets of the diabetic milieu that passively increase hemodynamic resistance, our review proposes a paradigm shift, positioning biochemically altered erythrocytes as active, mechanically abrasive contributors that directly traumatize the renal microvasculature.

Red blood cells (RBCs) or erythrocytes represent the vast majority of blood constituents, while circulatory system naturally distributes them throughout the body, providing access to various tissues and making them a valuable tool for the assessment of health status [9]. Erythrocytes are constantly exposed to environmental stimuli to which they react though metabolic adaptations, making RBC-related biomarkers useful in the diagnosis and monitoring of DM and its complications [9,10]. Additionally, erythrocyte’s membrane is the best studied plasma membrane and a reliable marker of systemic metabolism as RBCs lack a nucleus and organelles [11]. Given their vast abundance, combined with the minimally invasive nature of their collection and isolation, erythrocytes represent a useful, cost-effective choice for research and diagnostic purposes [9].

Building upon this diagnostic potential, the present review endeavors to systematically examine the alterations in membrane proteins, lipids and overall rheological behavior, aiming to establish a comprehensive pathophysiological framework that underscores the critical need for advanced multi-omics profiling in the early detection of DKD.

## 2. Molecular & Biochemical Alterations of the RBC Membrane in Diabetes

### 2.1. Overview of the Normal Erythrocyte Membrane Architecture

The erythrocyte membrane architecture is a highly intricate structure, conventionally described as a fluid lipid bilayer of phospholipids and cholesterol anchored to a two-dimensional elastic network of skeletal proteins. This cytoskeleton is tethered to the bilayer through specific interactions with the cytoplasmatic domains of transmembrane proteins. Together with oligo- and polysaccharides, these components form the glycocalyx [12,13], providing RBCs with unique mechanical properties, structural stability under shear stress and necessary deformability for capillary transit [11,14].

The membrane proteome consists of hundreds of proteins that facilitate transport, adhesion, signaling and structural integrity [12]. According to their function, membrane proteins can be classified into three major categories: cytoskeletal proteins (e.g., spectrin, actin, protein 4.1), integral structural proteins (e.g., band 3, glycophorins) and anchoring proteins (e.g., ankyrin, protein 4.2) [15].

The structural core of the erythrocyte cytoskeleton is the spectrin-actin junctional complex, where spectrin heterodimers self-associate into tetramers to form a hexagonal lattice [12,13]. This network is the primary determinant of membrane elasticity, with its stability modulated by the phosphorylation of protein 4.1 by protein kinase C (PKC), protein kinase A (PKA) and calmodulin, allowing for dynamic stiffness adjustments during transit [12,15]. Structural integrity is further maintained by the ankyrin-based complex, which bridges the spectrin cytoskeleton to the cytoplasmic domain of band 3, the major integral protein and anion exchanger [11,12,15,16]. Additionally, glycophorins, particularly glycophorin A (GPA) provide a negative surface charge through sialic acid residues, creating electrostatic repulsion that prevents RBC aggregation and endothelial adhesion [15].

Lipids constitute the other major structural component of the erythrocyte membrane, organizing into a 5 nm thick bilayer dictating membrane fluidity, permeability and protein function. Although under homeostatic control, lipid composition responds to metabolic and dietary shifts, directly impacting membrane rheology [16,17].

Erythrocytes membrane is composed primarily of glycerophospholipids, specifically phosphatidylcholine (PC), phosphatidylethanolamine (PE), phosphatidylserine (PS), phosphatidylinositol (PI), along with sphingomyelin (SM) and cholesterol, alongside minor lysolipid and glycolipid fractions. While lipids roles remain partially characterized, evidence suggests they are implicated in regulating membrane protein activity and maintaining the cellular oxidoreductive balance [11].

Despite shared amphipathic properties, glycerophospholipids and sphingolipids possess distinct structural backbones [16]. Glycerophospholipids consist of a glycerol backbone with fatty acyl chains at sn-1 and sn-2, and a polar head group linked via a phosphate group at sn-3. PC dominates eukaryotic membranes, typically containing one cis-unsaturated fatty acyl chain [16,18]. Notably, plasmalogens, a unique glycerophospholipid subclass, appear to play a pivotal role in cellular protection. Their structure is marked by the presence of a vinyl-ether bond at the sn-1 position, rather than the standard ester bond, alongside an ester bond with a poly-unsaturated fatty acid in the sn-2 position and a polar head group at sn-3. This configuration promotes tighter membrane packing and enhanced stability. Crucially, while their specific structural role is debated, plasmalogens exhibit antioxidant functions as the vinyl-ether bond is susceptible to oxidation, allowing plasmalogens to scavenge reactive species. This protects integral unsaturated fatty acids (UFA) and lipoproteins from oxidative damage, to preserve the overall integrity of the membrane [11].

SM, the principal outer-leaflet sphingolipid, is built upon a sphingosine backbone. Its amino group binds a long-chain fatty acid to form ceramide, with the polar head group attached at the sn-1 hydroxyl [16,18].

The lipidome exhibits vast chemical diversity and variability in molar ratios, which fundamentally dictates collective membrane behavior. The relative head-group size and fatty acid profile govern phospholipid packing, bilayer thickness and spontaneous curvature [17,19,20]. Approximately 40% of fatty acid tails are unsaturated, influencing fluidity, permeability and channel kinetics, while also serving as lipid signaling precursors [16,20]. Consequently, altered fatty acid composition compromises cellular responsiveness to stimuli, making RBC lipidome analysis a valuable diagnostic window into systemic metabolic health and disease pathogenesis [20].

Cholesterol is the predominant non-polar membrane lipid [18]. Its rigid steroid nucleus, composed of four hydrocarbon rings, functions as a stabilizing agent for the membrane phase. Cholesterol, in its free form, accounts for up to 50 mol% of the total membrane lipid content. Lacking LDL receptors, mature RBCs rely entirely on lipoprotein metabolism for cholesterol acquisition, with exchange rates regulated by membrane SM and circulating lecithin-cholesterol acyltransferase (LCAT) activity [16,19].

The specific lipid composition of the plasma membrane is a critical determinant of intrinsic curvature of the membrane, bilayer fluidity and cellular deformability [11,17]. This composition is highly dynamic, fluctuating with diet, circadian rhythms and the cell cycle, thus serving as a biomarker of metabolic status. Moreover, red blood cells possess the capacity to sense lipid levels, either directly or via metabolic precursors and by-products, and adjust membrane physical properties to maintain homeostasis [17]. In contrast to other cell types, erythrocytes maintain a high cholesterol-to-phospholipid ratio approaching 50%. This elevated ratio holds profound functional implications as it modulates the activity of specific membrane transporters and facilitates efficient gas exchange [21].

Within biological membranes, specific regions enriched in sphingolipids and cholesterol spontaneously segregate to form lipid rafts. These dynamic microdomains, ranging from 10 nm to 200 nm in diameter, serve as organizing centers for signaling and anchor GPI-linked proteins (e.g., CD55, CD59) [11].

Finally, lipids are not symmetrically distributed across the bilayer; this asymmetry arises from biophysical properties that dictate the rate of spontaneous transbilayer movement. While size, charge and head group polarity influence passive diffusion, specific transport mechanisms actively maintain this gradient. Scramblases, activated by Ca^2+^ function in an ATP-independent manner, to randomize phospholipid distribution. In contrast, ATP-dependent aminophospholipid translocases selectively pump PS and PE from the outer to the cytosolic leaflet. This active transport creates an imbalance between leaflets that contributes to membrane bending and curvature. This asymmetry has critical functional consequences; the confinement of PS to the inner leaflet prevents it from serving as a susceptibility signal for eryptosis or a catalytic surface for the coagulation cascade. In the normal RBC membrane, PC and SM are predominantly located in the outer leaflet while PS, PE and PI are sequestered in the inner leaflet. The transbilayer distribution of cholesterol remains dynamic and is not strictly asymmetric [16,18]. Furthermore, a variety of ion carriers including the Na/K ATPase and the Na/H exchanger (NHE1) are embedded within this lipid matrix, actively maintaining the ionic gradients essential for cell volume regulation [11].

### 2.2. Membrane Proteins Modifications

Mature erythrocytes lacking nuclei and repair machinery, serve as long-term reservoirs for Amadori products and advanced glycation end-products (AGEs) during their 120-day lifespan [22]. In the diabetic milieu, chronic hyperglycemia and systemic oxidative stress alter the secondary structure of membrane proteins drastically reducing solubility and structural flexibility linked to microvascular injury initiation [23].

The accumulation of damaged proteins is further exacerbated by the collapse of the erythrocyte’s internal clearance systems. Oxidative stress downregulates endogenous antioxidant defenses, allowing highly reactive lipid peroxidation end-products, such as 4-hydroxynonenal (4-HNE), to accumulate. These byproducts exert a direct, toxic inhibitory effect on erythrocyte’s 20S proteasome, suppressing its chymotrypsin-like activity [24]. This proteolytic failure prevents the clearance of oxidized proteins, leading to widespread protein carbonylation [25]. The degree of membrane protein carbonylation correlates directly with the clinical progression of diabetic microvascular complications [26,27]. Furthermore, oxidative attack on protein thiol (-SH) groups, induces degradation and high-molecular mass aggregation [28].

Advanced proteomics identify the RBC cytoskeleton’s foundational pillars (spectrin, ankyrin, protein 4.1) as primary non-enzymatic glycation targets [22]. Glycation induces rigid interhelix cross-links in spectrin, preventing the obligatory unfolding of spectrin repeats required for cellular deformation and causing pathological condensation of the actin-spectrin network [29,30]. This rigidification directly impairs erythrocyte transit through narrow glomerular capillaries. While antioxidants like N-acetylcysteine can partially recover spectrin distribution [31], the diabetic environment simultaneously reduces membrane-bound ankyrin and displaces PKC to the cytosol, further destabilizing the bilayer and stiffening the cell [32,33].

Band 3, the primary membrane-cytoskeleton anchor, is a major target of the diabetic milieu. Oxidative stress triggers its disulfide cross-linking and tyrosine hyperphosphorylation, severing its connection to the spectrin-ankyrin matrix [34]. Additionally, glycated intracellular hemoglobin excessively cross-links with the cytoplasmic domain of Band 3, forming stable, pathological aggregates that eradicates the membrane’s viscoelasticity [35,36,37].

Beyond the cytoskeleton, glycation directly warps the physical architecture of critical integral membrane proteins. For instance, glycation-induced conformational shifts in the GLUT1 outer domain increase its activation energy, severely restricting cellular glucose influx [38,39]. Consequently, highly aberrant expression levels of GLUT1, GLUT3 and other transporters are consistently noted in diabetic membranes as a result of long-term glycemic exposure [40,41,42].

Finally, glycation of flotillin-1 and flotillin-2 disrupts cholesterol-rich lipid rafts, coupling cytoskeletal failure with pathological lipid bilayer reorganization [22]. Glycation also structurally inactivates CD59, a critical membrane regulatory protein, leaving the diabetic erythrocyte highly susceptible to complement-mediated lysis [43]. Similarly, oxidative damage to the membrane surface acts as a primary driver for the detachment and shedding of sialylated glycoproteins, such as glycophorins, into the plasma, effectively stripping erythrocytes of their protective negative surface charge and promoting abrasive adhesion to the glomerular endothelium [44].

### 2.3. RBC Membrane Lipid Alterations

The erythrocyte lipid bilayer is vulnerable to the intense oxidative stress generated by the diabetic environment, which overwhelms endogenous antioxidant defenses such as erythropoietin [45]. This oxidative environment drives severe lipid peroxidation, evidenced by the marked accumulation of malondialdehyde (MDA) and highly reactive end-products like (4-HNE) localized to the outer plasma membrane leaflet [24,25,28,33,46]. The structural integrity of specific fatty acids is actively compromised; for instance, oxygen free radicals abstract hydrogen atoms from linoleic acid (LA) in the inner lipid leaflet, forcing a double-bond shift that generates conjugated linoleic acid (CLA), a process that strongly correlates with HbA1c levels [47]. Furthermore, membrane cholesterol undergoes profound oxidation, yielding toxic minor products such as 3-cholesten-6-one and 7-oxocholesterol, which further destabilize the membrane architecture in a manner dependent on the severity of hyperglycemia [48]. Ultimately, the uncontrollable generation of MDA induces the formation of lipofuscin, creating polymeric complexes that rigidly cross-link membrane glycoproteins and phospholipids together [49].

Beyond oxidative degradation, the diabetic erythrocyte suffers from pathological lipid remodeling and cholesterol loading. Diabetic membranes exhibit a significantly elevated percentage of cholesterol and increased ratio of saturated fatty acids (SFAs) to UFAs [49,50]. This cholesterol accumulation, which worsens as diabetes progresses to complications like DKD disrupts the normal packing of lipids [33,51,52]. At the molecular level, these alterations manifest as disordered phospholipid head groups, a restricted interface region, and highly ordered lipid hydrocarbon chains, which collectively create a much stiffer membrane interior [23]. In vitro depletion of this excess membrane cholesterol has been shown to reduce free radical-induced damage, proving that pathological cholesterol loading actively lowers the threshold for lipid peroxidation [49].

This rigidification is severely compounded by a massive shift in the membrane’s fatty acid profile. The diabetic milieu actively depletes the membrane of its flexible, protective polyunsaturated fatty acids (PUFAs), drastically lowering the omega-3 index and elevating the omega-6 to omega-3 ratio [53]. While PUFAs normally exert renoprotective and anti-inflammatory effects, the pervasive oxidative stress in diabetes completely overwhelms these defenses, rendering them ineffective at protecting the microvascularate [54]. In their place, SFA, such as myristic, palmitic, and stearic acids are accumulated making the membrane stiffer [54,55]. Moreover, nervonic acid is also accumulated, indicating increased concentrations of rigidifying SM [49]. Interestingly, while beneficial long-chain PUFAs are annihilated, arachidonic acid levels paradoxically rise, providing an abundant substrate that is highly susceptible to further free radical attack [50].

Lastly, structural and oxidative damage to the diabetic erythrocyte culminates in a profound loss of transverse lipid asymmetry. Under physiological conditions, specific phospholipids are strictly segregated between the inner and outer leaflets. However, in diabetes, this organization collapses, leading to an increased overall proportion of SM and saturated phospholipids [56]. Most critically, a significant percentage of endogenous PS and PE are inappropriately flipped from the inner monolayer to the outer membrane surface [57,58]. This externalization of PS, which may be partially attributed to the inhibition of flippase activity by increased membrane-associated tubulin, acts as a potent destruction signal [59]. It renders the outer lipid leaflet less tightly packed and more disordered, leaving the membrane highly vulnerable to phospholipase A2 and initiating the cascade of programmed cell death known as eryptosis [46,57].

### 2.4. Pump Alterations: Ionic Imbalance and Transporter Dysfunction

The biochemical degradation of the erythrocyte membrane profoundly disrupts the function of its embedded ion pumps, fundamentally altering intracellular electrolyte homeostasis. The most consistently reported transport defect in the diabetic erythrocyte is the significant suppression of the sodium-potassium pump (Na+/K+-ATPase). Multiple studies demonstrate a drastic reduction in Na+/K+-ATPase activity under hyperglycemic conditions, a defect that strongly correlates with poor long-term metabolic control rather than acute glucose fluctuations [60,61,62]. This suppression reflects chronic metabolic remodeling akin to accelerated cellular aging and is distinctly linked to the progression of microvascular complications, including diabetic kidney disease and retinopathy [63,64]. While the majority of evidence points to a loss of function, the literature presents some functional paradoxes; certain cohorts exhibit no significant alterations or lack correlation with short-term glycemic markers [65,66]. A transient increase in pump activity, hypothesized to be an early, temporary compensatory mechanism against osmotic stress has also been reported [52].

Calcium handling within the diabetic erythrocyte is similarly compromised, though the mechanisms of failure are highly complex. Elevated glucose exposure has been shown to significantly depress the activity of both Ca^2+^-ATPase and Ca^2+^/Mg^2+^-ATPase, leading directly to a loss of erythrocyte flexibility [67,68]. However, the susceptibility of these calcium pumps appears to depend heavily on the intracellular microenvironment. Several studies indicate that within fully intact erythrocytes, Ca^2+^-ATPase activity remains surprisingly preserved despite systemic hyperglycemia [66,69]. This preservation occurs because intracellular ATP physically shields the vulnerable lysine residues located near the enzyme’s catalytic site, effectively preventing the non-enzymatic glycation that would otherwise paralyze the pump [70]. This indicates that while the calcium extrusion machinery is biochemically vulnerable to oxidative damage, localized compartmentalization may offer varying degrees of in vivo protection.

As ATP-dependent pumps fail or struggle to maintain gradients, the diabetic erythrocyte experiences a pathological upregulation of alternative ion exchange mechanisms. Hyperglycemia specifically stimulates the activity of the sodium-hydrogen exchanger (NHE-1), driving an abnormal influx of ions that directly increases cellular volume and exacerbates osmotic fragility [60,71]. Concurrently, the activity of the sodium-lithium countertransport (SLC) system is significantly elevated in diabetic cohorts and correlates directly with HbA1c levels [66]. This profound shift in transmembrane ion handling, characterized by the simultaneous inhibition of primary active transporters and the hyperactivation of secondary exchangers, leaves the erythrocyte highly vulnerable to swelling, ionic toxicity, and eventual lysis within microcirculation.

## 3. Hemorheological Consequences: Aggregation and Deformability

In the setting of impaired glucose homeostasis and diabetes mellitus, alterations in the plasma membrane of erythrocytes are directly translated into profound rheological disturbances and consequently, functional failure [72,73]. Under physiological conditions, erythrocyte deformability is closely regulated by membrane composition, cytoskeletal organization, and internal cytoplasmic viscosity [11]. However, their mechanical adaptability is severely impaired due to hyperglycemic conditions and increased intracellular sorbitol [74]. In diabetic cohorts, assessment of erythrocyte’s deformation index, dynamic elasticity, and surface viscosity, utilizing ektacytometry and holotomography reveal a dose- and a time- dependent decrease of these parameters [75,76]. In type 2 diabetes mellitus (T2DM), erythrocytes are characterized by a lower surface area-to-volume ratio, indicating a pathological biophysical remodeling that significantly increases membrane tension and eliminates the capacity for elastic deformation [77]. Membrane’s rigidification is mechanically enforced by the translocation of hyperacetylated tubulin into the membrane [78]. Interestingly, apart from chronic metabolic stress, acute hyperglycemic spikes are also capable of severely compromising erythrocyte’s ability to adapt to the physical forces applied by blood flow, establishing reduced deformability as a promising biomarker for disease progression [77,79,80].

This loss of cellular flexibility acts as an important driver of pathological aggregation and hemodynamic sludging within the microcirculation [81]. Healthy RBCs typically undergo reversible adhesion to form organized rouleaux; however, exposure to hyperglycemic environments cause diabetic erythrocytes to clump into disordered, irregular, and highly branched aggregates [75]. Plasma fibrinogen and higher saturation levels in membrane phospholipids further contribute to this pathological aggregability noted in diabetes mellitus [56]. These stiffened, highly adhesive erythrocyte aggregates fail to disperse even under high shear stress, blood viscosity rises significantly at low shear flows, perturbating capillary transit [7,13]. Microvascular flow disturbance is not attributable only to erythrocyte membrane, but is also compounded by the diabetic plasma milieu, where concomitant dyslipidemia and systemic inflammation confine the vulnerable erythrocyte in a highly resistive rheological environment [82].

Crucially, the biophysical alterations that make erythrocytes pathological to the microvasculature are not uniform across all diabetic states and are far more complex than simple physical occlusion [83]. Normal erythrocytes release ATP, stimulating nitric oxide production by adjacent endothelial cells, prompting vasodilation and facilitating their own passage [84]. In T2DM, insulin resistance and dyslipidemia make erythrocytes to trigger an arginase-driven oxidative stress pathway, reducing nitric oxide bioavailability and driving localized vascular dysfunction independently of HbA1c levels [83]. Moreover, vesiculation of erythrocytes enables the extension of these toxic events beyond the parent cells. Extracellular vesicles shed from the diabetic erythrocyte membrane, contain a pathogenic protein cargo, including arginase-1. These vesicles use altered surface glycoproteins and fuse with the vascular endothelium, actively propagating oxidative damage throughout the microvasculature [85].

The rheological defects that are noted in diabetes mellitus, exert important damage on target organs, as tissue perfusion is drastically reduced and localized hypoxia is induced, accelerating the occurrence of diabetic complications [86,87,88,89,90]. Specifically, erythrocyte properties serve as direct predictors for the development of diabetic retinopathy, where a progressive reduction in cellular deformability mirrors the deterioration from uncomplicated diabetes to proliferative disease [91,92]. Additionally, alterations in erythrocyte’s membrane induced by diabetes mellitus compromise the cell’s physical endurance, leading to profound osmotic instability and mechanical fragility [78,93]. In diabetes, as non-deformable, fragile erythrocytes circulate though narrowed, microangiopathic blood vessels, sheer mechanical stress causes them to rupture prematurely. This results in a constant, in vivo mechanical hemolysis which acts as a primary mechanism accelerating the onset of anemia in diabetic patients [75,94].

## 4. The Pathophysiological Bridge: From Altered Rheology to Kidney Injury

**Figure 1:**
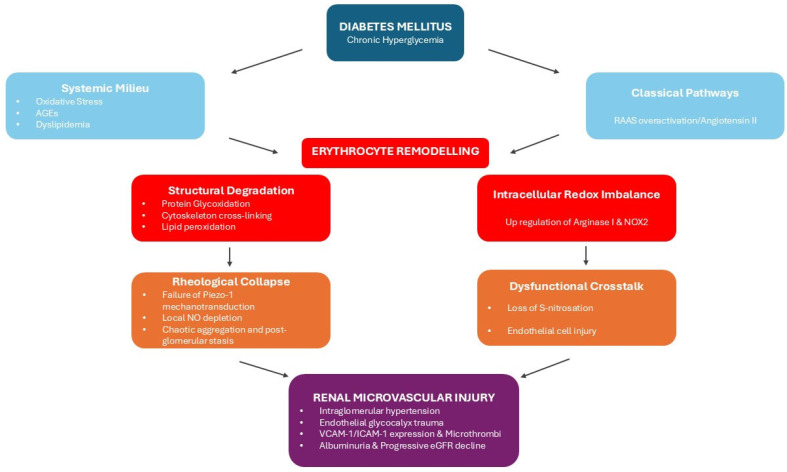
Integrative flowchart of the pathogenic cascade in erythrocyte-driven diabetic kidney disease: As illustrated in this flow diagram, chronic hyperglycemia drives the activation of classical DKD pathways and a systemic oxidative milieu, which synergistically induce profound erythrocyte membrane remodeling. This cellular degradation initiates a dual pathogenic cascade: (1) Rheological collapse, marked by loss of deformability and (2) dysfunctional crosstalk, wherein altered erythrocytes transfer oxidative stress directly to the endothelium. These parallel mechanical and molecular pathways converge to inflict severe renal microvascular injury, driving the progression of DKD.

While the biophysical alterations of the diabetic erythrocytes impair microvascular circulation systemically, the detrimental effects of altered rheology are most evident within the kidney vascular system (Figure 1). Kidneys rely on tightly regulated hemodynamics to maintain glomerular filtration. According to the Hagen-Poiseuille law, the already increased blood viscosity in diabetes mellitus, is significantly magnified as it reaches the efferent arterioles due to the process of post-filtration hemoconcentration [82]. When rigid, non-deformable erythrocytes and cellular aggregates arrive at this post-glomerular bottleneck, they precipitate a state of severe rheological impedance and microvascular stasis. This resistance to outflow creates a retrograde pressure gradient, inducing mechanically intraglomerular hypertension. This hemodynamically driven pressure exerts physical strain to the glomerular filtration barrier and provides a direct pathophysiological mechanism by which altered rheology due to erythrocyte membrane’s modifications in diabetes mellitus, may contribute to the onset of glomerular injury and albuminuria [82].

The breakdown of the protective erythrocyte–endothelial crosstalk further amplifies the destructive effects of the aforementioned mechanical obstruction. Under healthy conditions, the physical deformation of RBCs under shear stress activates mechanosensitive piezo-1 channels and triggers the release of ATP. The latter stimulates endothelial nitric oxide synthase (eNOS) to produce vasodilatory nitric oxide (NO). On the other hand, in diabetes mellitus, rigidified erythrocytes fail to deform and to activate this mechanotransductive pathway resulting in local depletion of NO [95]. This NO deficiency removes a critical vasodilatory factor, causing the renal microvasculature to yield to vasoconstrictive factors like endothelin-1, further increasing intrarenal vascular resistance and reducing overall renal blood flow [96].

A vicious cycle between rigid erythrocytes and glomerular endothelium injury is developed. Lack of bioavailable NO, removes the protective S-nitrosation of erythrocyte proteins, leaving them vulnerable to further oxidative damage and as a result to rigidification [95]. As these rigid, adhesive erythrocyte aggregates stall within the glomerular capillaries, they injure glomerular endothelial cells and remove the protective endothelial glycocalyx [96]. Disruption of the endothelial barrier and local hypoxia trigger the expression of adhesion molecules such as Vascular Cell Adhesion Molecule-1 (VCAM-1) and Intercellular Adhesion Molecule-1 (ICAM-1). These molecules trap circulating leukocytes and promote the development of microthrombi that occlude and obliterate the kidney microvascular network (Figure 1) [96].

Advanced clinical diagnostics provide strong evidence supporting this erythrocyte-driven model of kidney injury. Cross-sectional studies utilizing holotomography and critical shear stress assays demonstrated that altered erythrocyte deformability and increased aggregation strongly correlated with a declining in estimated glomerular filtration rate and the progression of diabetic kidney disease [97,98]. Specifically, quantification of erythrocyte aggregation at specific shear stress cutoffs provides a sensitive, point-of-care microvascular biomarker for renal impairment [98]. Additionally, while in healthy erythrocyte membrane profile enriched in polyunsaturated fatty acids normally exerts renoprotective and anti-inflammatory effects, persisting oxidative stress in diabetes overcomes these endogenous defenses and renders the membrane unable to prevent chronic kidney disease progression [54]. Ultimately, the progressive accumulation of uremic toxins and the compensatory release of reticulocytes, further compound the systemic rheological burden, highlighting that the biophysical failure of the erythrocyte acts as a driving mechanism of diabetic kidney disease pathogenesis (Figure 1) [92].

While the mechanistic basis of erythrocyte rheological impairment in diabetes is well-documented, direct clinical evidence correlating these specific biophysical alterations with the onset and progression of DKD remains remarkably sparse. Table 1 summarizes the key clinical studies [54,98,99,100,101] that have successfully bridged this gap, demonstrating significant associations between hemorheological parameters and established renal metrics. Despite these compelling associations, the current body of patient-based research presents certain methodological limitations. Most available studies rely mainly on macroscopic indices or evaluate isolated rheological parameters at a single point in time. While this establishes a clear critical link between erythrocyte dysfunction and renal impairment, it often fails to capture the complex, underlying molecular alterations driving this progression. To overcome these limitations, future clinical investigations must expand beyond static, macroscopic observations. Tracking the longitudinal biochemical and structural remodeling of the erythrocyte alongside DKD progression will be critical. Lastly, bridging the gap between descriptive hemorheology and its underlying molecular drivers is an essential step toward establishing reliable, mechanism-based biomarkers.

## 5. Limitations and Controversies

While the evidence presented strongly suggests an active role for erythrocytes in the pathogenesis of diabetic kidney disease, the proposed erythrocyte-centric framework must be critically contextualized within the broader landscape of diabetic microangiopathy and the limitations of current literature.

### 5.1. Alternative Mechanisms and Pathophysiological Synergy

The erythrocyte-centric model does not replace established classical mechanisms. Decades of research have thoroughly documented that DKD is driven by a complex interplay of metabolic (hyperglycemia, dyslipidemia) and hemodynamic factors in genetically susceptible individuals, leading to glomerular hyperfiltration, sterile inflammation mediated by cytokines and macrophage infiltration and fibrosis [5,102]. Rather than competing with these classical pathways, erythrocyte biophysical failure operates in a synergistic vicious cycle with them. Classical DKD mediators directly drive red blood cell structural failure, as angiotensin II has been shown to induce profound erythrocyte senescence, intracellular antioxidant depletion and pro-inflammatory membrane lipid remodeling via the angiotensin II receptor type 1 [103]. Simultaneously, diabetic erythrocytes act as active pathogenic potential contributors rather than passive bystanders of systemic toxicity. By up-regulating intracellular arginase I and NOX2, these structurally altered cells transfer significant oxidative stress to the vascular wall, precipitating and sustaining endothelial dysfunction [104].

### 5.2. Limitations of Current Evidence

Despite these mechanistic insights, the existing literature evaluating erythrocyte rheology faces distinct methodological limitations. A substantial portion of the clinical data derives from cross-sectional cohorts with relatively small sample sizes, which inherently limit statistical power and precludes the establishment of definitive causality. Furthermore, much of the foundational rheological knowledge relies on static in vitro models. While informative, these settings fail to accurately replicate the complex hemodynamic forces and mechanical shear stress present within the in vivo glomerular microcirculation. Additionally, previous molecular characterizations of the diabetic erythrocyte membrane have frequently been reductionist, often isolating single components rather than employing the comprehensive, integrated multi-omics approaches advocated in this review.

### 5.3. Controversies and the Scope of Current Literature

Even if direct contradictions to the erythrocyte-centric model are currently scarce, this absence must be interpreted critically. Historically, foundational DKD research has predominantly focused on classical biochemical markers and pathways and standard clinical trials and in vivo models rarely incorporate advanced hemorheological profiling. Because of this historical methodological “blind spot”, the current literature lacks targeted in vivo interventions capable of exclusively modulating erythrocyte deformability independently of systemic glycemic or inflammatory control. Consequently, the primary controversy in the field lies not in conflicting datasets, but rather in the interpretation of correlative data and specifically, delineating the exact timeline of whether erythrocyte biophysical failure strictly precedes or develops in parallel with early microvascular injury. This highlights the urgent necessity for future longitudinal trials specifically designed with integrated rheological endpoints.

## 6. Conclusions and Future Perspectives

Diabetes mellitus causes erythrocytes to undergo a significant transformation from highly deformable, protective oxygen carriers into rigid, potentially important pathogenic contributors of microvascular disease. The cumulative burden of mechanisms such as non-enzymatic protein glycation, lipid peroxidation and the dysfunction of ion pumps results in the loss of cells’ viscoelasticity. As these structurally altered cells circulate, their inability to deform and their increased propensity for aggregation precipitates microvascular stasis. In the tightly regulated kidney microcirculation, rheological abnormalities strain the glomerular filtration barrier while the loss of mechanotransductive nitric oxide signaling, and the release of toxic extracellular vesicles destroy the vascular endothelium. Therefore, the biophysical failure of red blood cells must be recognized not solely as a consequence of systemic metabolic dysregulation but also as a primary, active mechanism in the pathogenesis and progression of diabetic kidney disease.

**Figure 2:**
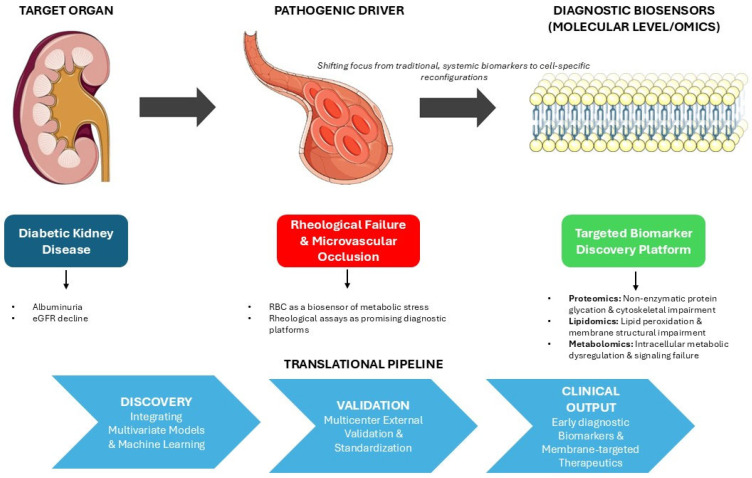
An erythrocyte-centric multi-scale translational framework for biomarker discovery in diabetic kidney disease. While the target organ (kidney) manifests late clinical endpoints, a pivotal pathogenic mechanism is microvascular rheological failure, mechanistically rooted in the molecular architecture of the erythrocyte. Applying targeted multi-omics profiling establishes the red blood cell as a high-fidelity diagnostic biosensor. Bridging the translational gap requires the integration of these molecular signatures via multivariate machine learning models and rigorous multicenter validation, ultimately yielding early diagnostic biomarkers and membrane-targeted therapeutic targets.

Recognizing this erythrocyte-driven pathophysiology underlines a critical limitation in current clinical practice, which relies mainly on systemic glycemic markers like HbA1c to predict microvascular complications. Future translational and clinical research should focus on utilizing the erythrocyte itself as a high-fidelity, integrated biosensor of metabolic stress. While rheological assays offer promising diagnostic platforms, mapping the precise molecular architecture that orchestrates the noted biophysical collapse could provide early biomarkers of diabetic complications. Advanced multi-omics approaches could assist in further understanding the erythrocyte pathology, shifting the focus from generalized oxidative stress to the exact molecular reconfigurations occurring within the cell (Figure 2). As mature erythrocytes lack active protein synthesis and turnover machinery, their proteomic, lipidomic and metabolomic profiles serve as a permanent, cumulative archive of the systemic diabetic milieu throughout their lifespan.

Applying a comprehensive multi-omics framework allows researchers to decode the specific rheological failure of the erythrocyte to an unprecedented resolution. Proteomic analysis enables the mapping of exact sites of tubulin hyperacetylation and spectrin cross-linking that injure the cytoskeleton, while metabolomics can trace the intracellular depletion of nitric oxide and the toxic accumulation of polyol pathway intermediates. Within this integrated landscape, the lipidomic profiling of the erythrocyte membrane remains a particularly crucial area of investigation. Mechanical rigidity and endothelial toxicity of diabetic erythrocytes are dependent on cholesterol loading, PUFAs depletion and the pathological externalization of specific phospholipids; consequently lipidomic analysis can identify the exact structural remodeling that precipitates microvascular occlusion. Furthermore, the integration of lipidomic data with alternative omics, such as proteomics and metabolomics, can significantly enhance these datasets and aid in their interpretation, revealing how such changes reflect and impact the pathogenesis of diabetic kidney disease. In this context, applying multivariate models and machine learning approaches can achieve high discriminatory diagnostic performance in discovery cohorts. By profiling these complex molecular signatures across different stages of diabetic kidney disease, early, diagnostic biomarkers long before clinical albuminuria or estimated glomerular filtration rate can be developed (Figure 2). However, translating these findings into clinical practice faces a persistent gap, often attributed to limited cohort sizes, insufficient statistical power, or the intrinsic biological complexity of metabolic regulation. Proposed biomarkers may fail because early studies do not adequately account for bias, pre-analytical variability, analytical uncertainty and context-dependent performance that emerge during validation and clinical development, highlighting structural weakness in study design and interpretation rather than isolated technical shortcomings. Ultimately, the utility of omics data depends on analytical robustness, biological interpretability and alignment with clinical decision-making. There is a pressing need for greater use of standardized references, protocols and quality control procedures to improve reproducibility. External validation is widely recognized as a critical step in biomarker discovery, meaning validation cohorts must be multicenter. Characterizing these integrated omics profiles will not only refine our prognostic capabilities but also unveil highly specific therapeutic targets aimed at stabilizing the cell membrane, preserving cellular deformability, and thereby halting the progression of diabetic kidney disease (Figure 2). Moving forward, true progress in establishing the erythrocyte as a clinical biosensor will depend on tighter integration among discovery scientists, clinicians, laboratory professionals and regulatory stakeholders.

## Figures and Tables

**Table 1:** Direct clinical evidence correlating erythrocyte structural and rheological alterations with Diabetic Kidney Disease progression.

Reference	Study Population	Erythrocyte Parameter	Clinical Metric	Key Findings
Brown et al. 2005 [99]	n= 57 T2DM patients [(stratified by renal function: normal, renal insufficiency, end-stage kidney disease (ESKD)] and n = 21 matched non-diabetic controls	RBC deformability	Serum creatinine and clinical CKD staging	RBC deformability significantly correlated with serum creatinine in T2DM patients with renal impairment (r = 0.43, *p* = 0.02). Deformability was significantly lower in early T2DM compared to non-diabetic controls (*p* = 0.0005)
Chung et al. 2018 [100]	n = 421 T2DM patients (stratified by Critical Shear Stress tertiles to compared DKD vs. non-DKD)	RBC aggregability (measured via Critical Shear Stress-CSS)	Risk and presence of DKD	CSS was significantly elevated in patients with DKD compared to those without DKD (*p* < 0.001) The highest CSS tertile was independently associated with DKD risk, robust to multivariate adjustment (age, sex, DM duration, hypertension, hemoglobin). Established a clinical cut-off value of CSS ≥ 310 mPa for indicating DKD presence
Lee et al. 2019 [101]	n = 470 (n = 248 T2DM patients and n = 222 pre-diabetics) stratified by uACR and eGFR stages.	Erythrocyte deformability (Elongation Index- EI), integrated into a composite hemorheological index (Fibrinogen x Erythrocyte Sedimentation Rate (ESR)/EI).	Urinary Albumin-to-Creatinine Ratio (uACR), eGFR, and presence of microalbuminuria.	The composite index was an independent predictor of uACR in multiple regression analysis, adjusted for confounders (β = 0.01, *p* < 0.001) Significant differences were observed across all eGFR-classified CKD stages Demonstrated strong predictive value for microalbuminuria prevalence with a ROC AUC of 0.762 (Sensitivity: 74.5%, Specificity:63.1%).
Park et al. 2022 [98]	n = 378 patients with T2DM (stratified according to the KDIGO 2012 risk classification zones)	Erythrocyte aggregability (Critical Shear Stress- CSS)	Clinical DKD diagnosis integrating both eGFR and uACR standard criteria.	CSS successfully identified DKD, concurring with integrated KDIGO standards. High-Risk Detection (Model 1): Differentiating DKD-positive from DKD-negative yielded 100% sensitivity and 77.8% specificity. Moderate-Risk Detection (Model 2): Expanding to include the orange risk zone yielded 75% sensitivity and 72% specificity.
George et al. 2026 [54]	n = 893 participants (n = 290 with T2DM and n = 603 at high risk for T2DM; 15.6% presenting with comorbid T2DM and CKD).	Erythrocyte membrane fatty acid composition	Prevalent CKD (defined as eGFR < 60 mL/min/1.73 m^2^ and/or UACR > 3 mg/mmol)	A higher RBC lipogenic index was strongly associated with odds of prevalent CKD (OR = 2.73, 95% CI [1.22–6.12], *p* = 0.015). Higher levels of total n-6 PUFAs and specific linoleic acid in the membrane were associated with odds of CKD (OR = 0.86, *p* = 0.025 and OR = 0.81, *p* = 0.035, respectively).

## Data Availability

No new data were created or analyzed in this study. Data sharing is not applicable to this article.

## Abbreviations

The following abbreviations are used in this manuscript:

DKD	Diabetic Kidney Disease
DM	Diabetes Mellitus
CKD	Chronic Kidney Disease
eGFR	Estimated Glomerular Filtration Rate
RBCs	Red Blood Cells
PKC	Protein kinase C
PKA	Protein kinase A
GPA	Glycophorin A
PC	Phosphatidylcholine
PE	Phosphatidylethanolamine
PS	Phosphatidylserine
PI	Phosphatidylinositole
SM	Sphingomyelin
UFAs	Unsaturated Fatty Acids
LDL	Low Density Lipoprotein
LCAT	Lecithin-cholesterol acyltransferase
NHE1	Na/H exchanger
AGEs	Advanced Glycation End-products
4-HNE	4-hydroxynonenal
MDA	Malondialdehyde
LA	Linoleic Acid
CLA	Conjugated Linoleic Acid
SFA	Saturated Fatty Acids
PUFA	Polyunsaturated Fatty Acids
SLC	Sodium-lithium contransporter
eNOS	Endothelial nitric oxide synthase
NO	Nitric oxide
VCAM-1	Vascular Cell Adhesion Molecule-1
ICAM-1	Intercellular Adhesion Molecule-1
ESKD	End-Stage Kidney Disease
CSS	Critical Shear Stress
EI	Elongation Index
ESR	Erythrocyte Sedimentation Rate
uACR	Urinary Albumin to Creatinine Ratio
